# Epidemic Jaundice Montgomery County, Pa.

**Published:** 1855-07

**Authors:** Hiram Corson


### MEDICAL EXAMINER.

NEW SERIES.—NO. CXXVII.—JULY, 1855.

### ORIGINAL COMMUNICATIONS.

### *Epidemic Jaundice in Montgomery County, Pa.*

By Hiram Corson, M.D.

While I do not think it necessary for every one who meets with a few cases of severe disease, even very unusual ones, to make them the bases of a *theory*, and from this limited experience to lay down rules of practice applicable to other cases, it is certainly the duty of all, to record facts and observations for future generalization, by those fitted for the work. A brief notice of some cases of jaundice which occurred in my practice in the latter part of last year, may, therefore, not be uninteresting. On the 8th Sept., called to No. 1, a healthy Irish woman, aged 25, pregnant, at or near seventh mo., with her third child. Found her deeply jaundiced; urine in a deep vessel resembled strong infusion of coffee; linen steeped in it becomes tinged a bright yellow; stools white; great soreness around the fore part of the body, pit of stomach, and margin of the ribs, while the *pain* was entirely confined to a spot about *an inch above the umbilicus*; appetite almost lost, but still drags herself about her work. She lives in a low place, near the river Schuylkill, and has been much worried for two weeks nursing her husband with dysentery. Ordered calomel and rhubarb to move the bowels, blue mass moderately every day, with occasional laxatives. On 15th slight labor pains commenced, which, in defiance of every effort to arrest them, increased so as to produce delivery on the 18th of a feeble child, which lived but about an hour. Up to this time the yellow color of the skin was as deep as I have ever seen it in any patient; it then began to subside, and in a few weeks the health and color were re-established; since quite well. This was in an ague district, but there was hardly a case of intermittent fever in that region during the last year, which is remarkable from the fact that there are many unacclimated persons annually engaged at the furnaces in the neighborhood.

No. 2.—Sept. 26. Miss B. C., aged 6 years, on high ground, two miles nearly from No. 1. Well marked case; rhubarb and magnesia, in small doses for a few days, relieved the symptoms, and in two weeks she was pretty well.

No. 3.—Sept. 26. Miss B., aged 18, has had for a few days chilliness, pains in bones, and other feelings which she thought heralded ague. I found skin and eyes yellow; urine like coffee; stools white, and several in 24 hours; pain just above navel; soreness on margins of ribs and at pit of stomach; still moving about her work. A few cathartic pills, some cream of tartar as drink, and she convalesced in a few days. High ground, ¼ mile from No. 2.

No. 4.—Mrs. C., aged 27, at 7th mo. of first pregnancy. Has been complaining for nearly two weeks of chilliness, pain in bones, loss of appetite, soreness of abdomen, &c. Now, Oct. 3d, jaundiced symptoms present; much pain in epigastrium; frequent vomiting. On 5th, pains like those of labor began, and continued to recur from time to time until the 8th, when she was delivered of a small feeble child, that lived but two or three hours. So great was the suffering in the region of the liver that venesection, cups, &c., were used before delivery. After confinement the liver could be felt enlarged and tender to the touch. She was very ill for eight days after, and then gradually convalesced; quite well ever since. The pulse generally about 60.

No. 5.—D. S., healthy, strong man, aged 30. Oct. 15th. Has been poorly, with "pain in bones, no appetite, bad feelings" for a week. Color of the skin alarmed him, and I was sent for; pulse 64; tongue natural in appearance; all the symptoms of confirmed jaundice; has been quite sore in hypochondriac region, but now only complains of pain at navel; has already taken two doses of purgative medicine; convalesced in about a week.

No. 6.—D. F., aged 13. Oct. 14. Has been poorly since last of September; for a week has been jaundiced, stools white, &c., &c.; has taken no medicine, and as I was called to see the mother and he is said to be better, I gave him no medicine, and he continued to improve, and in a week more was well, and has better health this spring, 1855, than he has had before; took no medicine of any kind; pulse 64.

No. 7.—Mrs. F., aged 35, and mother of No. 6, was "sore and stiff" for nearly two weeks before she saw the change in the skin, fæces and urine. It has been nearly 10 days since she has had confirmed jaundice, and the disease is abating, though she has taken no medicine, but she is alarmed to-day on looking at her urine. It is not so high colored as it has been, but is yellow and almost thick with ropy mucus. A lump can also be felt over the lower orifice of the stomach of the size of a medium orange, which is slightly tender to the touch; but for these considers herself getting well; pulse 64, and complains much of a taste like the smell of rotten eggs when eructations occur. Ordered a cathartic and some diuretics; convalesced.

No. 8.—L. S., aged 45, strong man, has had the disease strongly marked for a week; had pain nowhere, except *just above the navel*, although there was *soreness* all around epigastric and hypochondriac regions; no apparent loss of appetite, but always felt bad after eating, and several times threw up his food, but without much sickness; had taken epsom salts. Ordered rhubarb and magnesia; was well in about two weeks.

No. 9.—J. K. C., aged 18, active boy, has had pain in stomach; chills, &c., for a week; taste like that of hard boiled eggs, when he eructates; pulse only 60; no thirst; does not miss a meal, but is not hungry; jaundice well marked; mild purging relieved him in two weeks.

No. 10.—Miss M. L., aged 17. I was sent for on account of the deep yellow color of skin and eyes; has been intensely yellow for a week, but as she was a hired girl and made no complaint, she was left without medicine; complains scarcely at all of soreness or pain, even on pressure, yet the stools are chalky; urine like coffee, &c. Put her on the use of podophylline, first as a purgative, and afterwards as an alterative; convalesced in about three weeks. Although there was such an absence of suffering in this case, I thought I could plainly feel an enlarged liver.

No. 11.—Mrs. M. F., same house with the above, aged 47; well marked case; convalesced in two weeks.

No. 12.—Mrs. Mc., aged 36, married, pregnant at 7½ month with 7th child. Nov. 13. Has had giddiness and dulness in head for about one week; all the symptoms of the disease strongly marked; frequent eructations of the sulphuretted hydrogen taste; very giddy, so as to be compelled to lie down often. Her first symptoms were itching and burning in hands and feet at 4 o'clock every day. In about a week this left the hands, then the feet, and spread all over the body in the same way, at 4, P. M. This was accompanied with cough, which continued till some time in the night, and then they declined together. Although these symptoms occurred every day about 4 P. M., yet she was much worse every other day. Saw her Nov. 13; is very *giddy*, not sick; giddiness and yellow tinge of skin came on about a week since; taken one dose of quack pills; tongue nearly natural in appearance; no thirst; pulse 64; intensely yellow, quite costive; itching over the whole body, and but for the giddiness would be able to do her work. The pills which she had taken operated well in a few hours after my visit, and next day her pulse was at 80. Tuesday 21. Has taken quinine and rhubarb and blue mass since last visit; still thinks she is worse every other day; stopped all medicine to watch the case for a day or two. Wednesday 22d. She says that yesterday near evening, while up and about, pains and purging came on, so as to compel her to be on the chamber nearly constantly; at midnight took 20 drops laudanum, which relieved the pains, but the purging continued, and this morning on measuring the discharges I find a full gallon of whitish water with shreds and patches of white mucus such as occur in dysentery; swelling of the feet (which I forgot to mention) has disappeared. Ordered ext. bark. Friday 24. Feels pretty well, but no change in the jaundice. 27th. Delivered of a living child at 8th month. Dec. 5. Feels pretty well; yellowness slightly diminished; urine very high colored; stools white; has pain after eating, and yet the tongue looks quite natural; slowly recovered; nursed the child; milk did tinge the linen slightly I thought; child did well.

No. 13.—Mrs. J., aged 30, pregnant with third child, at 7½ months. Symptoms well marked. Labor came on in a few days; child and mother did well.

No. 14.—J. D., boy aged 10. Well marked case; Nov. 2d.

No. 15.—J. L., strong man, aged 26; poorly for two weeks but still at his work until to-day, Nov. 20; so wretched now he can work no longer. Chills; pain at navel; soreness in right side; thinks he has something like ague every other day; bowels moved twice nearly every night; pulse only 60 at any time until convalescence; tongue nearly natural. Bone-set infusion to vomit; bath; ex. bark. Dec. 1, convalescent.

No. 16.—C. L., healthy man. Nov. 14th, has been poorly nearly two weeks; now confirmed jaundice; is sore in epigastrium and margins of ribs, but refers all his pain to a spot an inch above the umbilicus; appetite impaired; slight thirst; pulse of natural fulness, but only 64 per minute; tongue slightly furred. Nov. 16, has been well purged; feels better, but pulse is now only 56 per minute; convalescent in a few days (aged 45).

No. 17.—Miss B. C., aged 5, not well for nearly two weeks; mother thought she had something like "dumb ague;" is now deeply jaundiced; capricious appetite; tongue nearly natural; playing almost as usual; pulse 66; well in two weeks.

No. 18.—T. L. a healthy carpenter, aged 22. Nov. 13 felt drowsy, and noticed a yellowness of skin; but about 17th had pain and soreness of abdomen, giddiness, shooting pain in the region of the heart; chills and fever several times a day; soreness of body on making a mis-step, or on lying down. I saw him on 21st; confirmed jaundice; pulse regular, and just 60 per minute; *tongue quite natural; appetite craving;* no enlargement of liver. Gave an aperient sol. of aloes and bi-carb. of soda every three hours, so as to purge daily. Nov. 25th, getting well. This case was doubtless recovering before I saw him, as the bitter taste had left him on the 19th, and appetite had become craving.

No. 19.—Mrs. L., aged 38, nursing her third child. Jan. 1st, 1855, has had chills and fever for two weeks nearly (never had ague.) Is intensely yellow; has a bitter taste, and everything she has eaten for many days has made her feel sick, but she has not vomited. She has thirst, but her tongue is clean and her pulse natural—72 per minute. Ordered sulph. zinci and ipecac. as an emetic, followed by podophylline as a cathartic. In four days much improved and considered as convalescent; pulse still at 72.

No. 20.—H. S., aged 15, strong healthy boy. Saw him Jan. 15; has been poorly since Jan. 5; had nausea; soreness of abdomen; pain above navel, and vomited once on taking some medicine; strongly jaundiced, and *pulse only* 52; purged him well; convalesced in a few days.

No. 21.—Irish boy aged 5. Jan. 9th, well marked case; gave podophylline; it vomited and purged freely, and he began at once to convalesce.

No. 22.—Hired girl but a few weeks from Germany; aged 28; has been poorly for two weeks; strongly jaundiced; on compound cathartic pills; relieved.

No. 23.—Miss E. L., aged 24. Strongly resembled an intermittent fever, but resisted the treatment by quinine; the symptoms of jaundice developed and then yielded to purgatives.

No. 24.—Miss H., aged 3 years; quite poorly for a week; all the well marked symptoms of jaundice; pulse 60; convalesced in a week.

No. 25.—Mrs. H., well marked case; sick two weeks, but has continued at her work. Treated by purgatives.

No. 26.—Mrs. L., aged 50; chills and fever; finally jaundice; cathartic every two or three days; convalesced.

No. 27.—Mrs. E. L., aged 48; quite a severe case; tried Dr. Physic's alkali and brandy medicine; produced severe vomiting; mild cathartics did well; pulse 56.

No. 28.—Mr. S. aged 26, shoemaker. Saw him Jan. 25; walked to my office. Five days since he first noticed his skin and eyes yellow; had felt well before, except a little uneasy for a couple of hours after eating; has vomited on two or three occasions; has had heart-burn and hiccups. Took a glass of brandy to sweat himself, but it vomited him severely; has no pain or soreness in the abdomen; is quite yellow. Ordered ap. sol. to move bowels gently every day. Convalesced speedily.

Dr. Leedom says, in a note to me, that he "had three cases of jaundice at Barren Hill (the infected district.) One was in an old man of 60 years; another a stout, robust man of 45; the third a man of 26." Besides these 31 cases, there were 7 who did not apply to any physician, or were prescribed for by some one in the neighborhood, and of which I have no account; these were all well marked cases, and known to me; doubtless there were other mild cases. All the cases save two were on the high ground along the Germantown and Reading Turnpike Road, and most of them within a space of less than half a mile; twelve cases occurred in nine families within a few yards of each other, and all at nearly the same time. There were twenty males and eighteen females. Of the males there were married 11; unmarried 9; two were over 70 years, four from 50 to 60, two from 40 to 50, three from 30 to 40, five from 20 to 30, one from 10 to 15, and one of 3 years. Of the females there were married 8, widows 2, unmarried 8; three over 40, three over 30, five from 20 to 30, four from 17 to 20, three from 3 to 5; four pregnant at from 6½ to 7½ mos., and had premature labors. The two children born at 7th month died within a few hours. Those at 8th month grew finely.

While many of the above cases were very severe, particularly those of the pregnant women, all recovered, and have been in good health ever since—a period of nearly six months. It may be said by some that these were cases of intermittent fever. In the course of my practice I have had many isolated cases of jaundice, and I can truly say that not even those from obstructions produced by gall stones were purer cases of jaundice than these. Intermittent fever has seldom appeared, even to the most limited extent, in the region where this epidemic prevailed most. In 1854 it did not prevail anywhere in this region. Now let us notice a few facts. Intermittent fever has pain in the back and head; furred tongue; thirst; quick pulse, &c. These cases were generally free from thirst; pulse slow, often from 50 to 60 in persons under age; tongue *natural in appearance*; no pains in the back; soreness in pit of the stomach and on margins of ribs; *pain* always at or an inch above the navel; ability to eat, but generally uneasiness after doing so.

Jaundice as an epidemic is new to me, but I offer these cases as food for thinking minds. Let us look at them all at once. Forty persons, of all ages and both sexes, and in good health, find themselves gradually becoming of a deep yellow color; pulse which was at 72 falls to 50 or to less than 60. The stools become white as chalk, and for the first few days there are several stools daily. The urine looks like coffee infusion; there is a pain near the navel, and soreness at pit of the stomach and on the ribs; there is nausea and vomiting at times; but mark, with all this evident change in the proper performance of the functions of the liver, in this *bilious* condition of the system, there is no thirst, no coat on the tongue, but on the contrary it is *natural in appearance.* The liver does not act, or, to use a common phrase, it is torpid, and yet the tongue is clean. There is pain and soreness in the abdomen, but the pulse is reduced fifteen or twenty beats per minute. It is evident that this torpor of the liver—this want of bile in the intestines, does not cause a change in the appearance of the tongue nor produce thirst. How must those persons be at fault who are always seeing a disordered condition of the liver in the furred tongue; and how pernicious is that error when it leads them to goad the system with that supposed specific for torpor of the liver, in order to clean the tongue. Is it not probable that the tongue is seldom affected except by disorders of the stomach alone?

The treatment pursued by me was by mild laxatives and purgatives. The podophylline was several times used as a substitute for the mercurials, or in other words as a purgative, and with good effect. Others were treated by the aperient solution made of aloes and bi-carb. soda, so highly recommended by Dr. Mettauer, of Virginia, in constipation. Occasionally emetics of bone-set, diuretics, podophylline or rhubarb, as an alterative, and venesection and cups in a few cases. I have heard of jaundice as an epidemic, but I believe it is rare, as I have not seen it noticed in works on practice, except a mere reference to it by Dr. Wood in his Treatise.

